# Hepatic Immunomodulatory Effect of Belatacept on the TNF-α/IL-6 Pro-Inflammatory Axis and Its Impact on Regulation of MiRNA-146a in Hepatic Injury

**DOI:** 10.1159/mpp/adpag021

**Published:** 2026-09-11

**Authors:** Doaa Khalid Ibrahim, Yassir Mustafa Kamal Al-Mulla Hummadi, Huda Jaber Waheed

**Affiliations:** a Department of Pharmacology and Toxicology, College of Pharmacy, University of Mosul, Mosul, Iraq; b Department of Pharmacology and Toxicology, College of Pharmacy, Al-Mustansiriyah University, Baghdad, Iraq

**Keywords:** Hepatic injury, Pro-inflammatory axis, MicroRNA-146a, Belatacept

## Abstract

**Objective:**

Acute liver injury is associated with a systemic cytokine release that determines the clinical prognosis. Although there are many hepatoprotective drugs, their unique immunomodulatory profiles are still unclear. This study evaluates the therapeutic potential of Belatacept versus Prednisolone on miRNA-146a expression and the pro-inflammatory cytokine cascade in a rat model of Concanavalin A-induced hepatic damage.

**Materials and Methods:**

The animal model of liver injury was induced in adult male Wistar rats by dividing them in 4 groups: normal controls, liver injury, prednisolone, and belatacept-treated groups. The serum levels of interleukin-6 (IL-6) and tumor necrosis factor-alpha (TNF-α) were used to determine the systemic inflammation and were measured by enzyme-linked immunosorbent assay. RT-qPCR was used to quantify the expression of hepatic miRNA-146a. Statistical significance was established by using one-way ANOVA (*p* < 0.05).

**Results:**

TNF-α and IL-6 levels in serum increased significantly, in response to inducing hepatic damage (*p* < 0.05). Alongside this, a notable dysregulation of miRNA-146a was detected. Belatacept treatment successfully inhibited the cytokine systemic response, bringing IL-6 down to levels that were statistically more significant than those attained by prednisolone (*p* < 0.01), while TNF-α reduction was comparable in both groups (*p* > 0.05). Additionally, Belatacept treatment effectively upregulates miRNA-146a levels, achieving a restoration profile which was statistically more significant than prednisolone (*p <* 0.01).

**Conclusion:**

These findings show that belatacept has stronger immunomodulatory action when compared with standard therapy. Belatacept is a promising new treatment option for liver-driven systemic inflammation which acts by inhibiting the TNF-α/IL-6 axis and modulating miRNA-146a.

**Highlights of the Study**

- Belatacept effectively silences the systemic tumor-necrosis factor-alpha and interleukin-6 cytokines axis.
- Immunomodulatory potency of Belatacept is higher than that of prednisolone.
- Belatacept restores miRNA-146a expression, promoting epigenetic homeostasis.
- Cytokine suppression and miRNA recovery via parallel distinct pathways.
- Belatacept is a multi-model candidate for liver-driven systemic inflammation.

## Introduction

Acute hepatic damage continues to be major problems in clinical hepatology due to swift occurrence of a systemic inflammatory response driven by tumor necrosis factor-alpha (TNF-α) and interleukin-6 (IL-6) [1, 2]. While corticosteroids like prednisolone remain the long-standing clinical standard for hepatic damage treatment [3], however, their nonspecific action and associated side effects underscore the need to evaluate novel, targeted immunomodulators that can effectively silence cytokine storm. To address this gap, attention has turned toward belatacept to investigate whether its proven immunomodulatory efficacy can offer a safer, tissue-protective alternative in acute hepatic injury. Belatacept is a recombinant Cytotoxic T-Lymphocyte-Associated protein 4 (CTLA4)-immunoglobulin fusion protein [4, 5]. Belatacept obtained FDA approval as calcinurin inhibitors (CNI)-sparing immunosuppressive agent in kidney transplantation in 2011 [6]. Despite clinical limitations, including a higher risk of early rejection or post-transplant lymphoproliferative disease [7], clinical data from renal transplantation show its distinct advantage in preserving renal function with significantly lower nephrotoxicity compared to CNI [8, 9]. However, the data related to belatacept use in liver transplantation remain limited and mixed [10, 11]. Pharmacological insights from kidney models suggest that Belatacept holds significant unrealized immunomodulatory potential for other tissue injuries [12], although its comparative efficacy against standard prednisolone in acute hepatic damage remains unexplored.

A critical molecular mechanism governing this inflammatory cascade involves microRNAs (miRNAs) which function as post-transcriptional regulators of immune response [13, 14]. Specifically, miRNA-146a serves as a key negative regulator of the TNF-α/IL-6 cytokine axis during hepatic stress [15]. Unravelling whether Belatacept exerts its hepatoprotective effects by modulating the miRNA-146a/cytokine axis can provide the basis for understanding its therapeutic role. Therefore, this study was undertaken to evaluate the immunomodulatory potential of Belatacept in a rat model of acute hepatic damage through a direct head-to-head comparison with prednisolone, specifically clarifying its capacity to suppress the TNF-α/IL-6 axis via the modulation of miRNA-146a expression.

## Materials and Methods

### Animals

Experimental Male Wistar rats weighing 175g + 25g were gotten from the Animal House of College of Pharmacy/Al-Mustansriyah University. The animals were maintained under standard circadian conditions, with ad libitum access to standard diet.

### Chemicals and Reagents

Concanavalin A (Con A) was purchased from (Sigma Aldrich Company, USA). Belatacept (Nulojix^®^) was purchased from (Bristol, USA), and prednisolone (Xilone^®^) was supplied (European Egyptian Pharmaceutical Industries, Egypt). Kits for the enzyme-linked immunosorbent assay (ELISA) determination of TNF-α/IL-6 were purchased from (SunLong Biotech company, China). RT-qPCR was performed using kits including that for miRNA extraction, Presto^®^ miRNA Purification Kit (Geneaid, Taiwan), Poly A Kit (OZ Biosciences, France), complementary DNA synthesis by EasyScript SuperMix kit (TransGen Biotech Co., China], and SYBR Green PowerUp Master Mix [Applied Biosystems, USA).

### Experimental Design

After an accommodation period, rats were divided in random manner into four groups with six animals in each of the following groups:

#### Normal Control group

The animals in the control group received a single intraperitoneal injection of normal saline on Day 0.

#### Liver Injury group

In this group, liver injury (LI) had been induced by single intravenous injection (15 mg/kg) of Concanvalin A on Day 0.

#### Prednisolone-Treated group

After inducing liver injury by Concanvalin A, treatment was initiated 24 hour-post induction by oral prednisolone (10 mg/kg/d for 2 weeks) on Day 1–14.

#### Belatacept-Treated Group

After inducing liver injury by Concanvalin A, treatment was initiated 24 hour-post induction by intraperitoneal injection of belatacept (60 mg/kg/w for 2 weeks) on Day 1 and Day 8.

### Sample Collection

Animals in normal control (NC) and liver injury (LI) groups were sedated with appropriate anesthetic drugs 24 h after the experiment began (Day 1) and sacrificed. After being drawn, blood samples were left to coagulate for approximately an hour at room temperature. Centrifugation at 3,000 rpm for 15 min was used to separate the serum, which was then kept at −20°C until biochemical and immunological tests. For miRNA study, hepatic tissues were promptly kept at −70°C. After 2 weeks (Day 14), this process was carried out once more for the other two groups (prednisolone-treated [PRED] and Belatacept-treated [BELA] groups).

### Enzyme-Linked Immunosorbent Assay

The concentrations of pro-inflammatory cytokines, specifically TNF-α and IL-6, were determined using specific rat TNF-α and IL-6 specific ELISA kits following the manufacturer’s protocol. Briefly, 10 µL of sample (rat serum) and 40 µL of sample diluent per well were added to pre-coated 96-well plates. After incubation and washing, biotinylated antibody and streptavidin-HRP conjugate were added. The reaction was visualized using 3,3′,5,5′-tetramethylbenzidine substrate and stopped with an acidic solution. The optical density was measured at 450 nm using ELISABiotek^®^ microplate reader. Cytokine concentrations were calculated from a standard curve and expressed as μg/L.

### MicroRNA Estimation by RT-qPCR

#### RNA Extraction and Poly A Tailing for miRNA Samples

Following the manufacturer’s instructions of the Presto™ miRNA Purification Kit, 20 mg of frozen hepatic tissue samples were used to isolate total small RNA. As soon as the miRNA was extracted, Poly(A) tailing was performed in a 20 µL reaction by mixing 10 µL of miRNA with 10 µL of master mix containing 10× Poly(A) Polymerase Buffer (2 µL), 10 mm ATP (1 µL), Poly(A) Polymerase (1 µL, 5 U/µL), and RNase-free water (7 µL).The reaction mixture was incubated in a thermal cycler at 37°C for 60 min to enable polyadenylation. Thermal deactivation was subsequently performed at 65°C for 20 min to terminate the reaction prior to downstream RT-PCR analysis.

#### Synthesis of Complementary DNA

We used the reverse transcription method. To guarantee specificity for miRNA analysis, the specific stem-loop primers for miRNA-155 and the internal control (U6 small nuclear RNA) were used (please add a reference for getting details of the method used). In summary, complete small RNA was reverse-transcribed in a final reaction volume of 20 μL, at 25°C for 10 min, 37°C for 120 min, and then at 85°C for 5 min for inactivating the enzyme. Total small RNA was isolated from frozen hepatic tissue samples according to the manufacturer’s protocol. Poly(A) tail has been added to miRNA immediately after extraction.

#### RT-qPCR or Quantitative Real-Time PCR

Using a StepOnePlus Real-Time PCR System and SYBR Green PowerUp Master Mix, the expression levels of miRNA-155 were measured. The complementary DNA template, forward and reverse primers, master mix, and nuclease-free water were all included in the 20 μL reaction mixture. The thermal cycling conditions used were, 10 min of initialization at 95°C, then 40 cycles (denaturation at 95°C for 15 s and annealing and extension at 60°C and 72°C, respectively, each one for 30 s). The comparative Ct method (2 ^−ΔΔCT^) was used to determine relative expression levels. Results were shown as fold change in comparison to the control group after data were adjusted to the expression of the endogenous reference gene U6 snRNA.

### Statistical Analysis

Mean ± standard deviation was used to represent the data. SPSS (version, 25.0) was used for statistical analysis. GraphPad Prism (version, 10) was used to create graphical representations and data visualization. One-way analysis of variance (ANOVA) was used to examine group differences, and Duncan’s multiple range test was used for post hoc comparisons. Hepatic miRNA expression levels and serum pro-inflammatory indicators (TNF and IL-6) were compared using a Pearson’s Correlation Coefficient (r) analysis. *p* values <0.05 were considered as statistically significant.

## Results

### Belatacept Suppressed TNF-α Levels in Liver Injury

Acute-phase assessment on 24 h after Con A-induced liver injury revealed a marked surge in serum levels of TNF-α in the LI group, showing 1.5-fold elevation over the NC group (*p* < 0.01). This cytokine release was markedly decreased after 2-week of therapeutic regimen. In particular, the BELA group experienced a 54% decrease in serum TNF-α levels, but the PRED group only experienced a 22% improvement, which is statistically insignificant (*p* > 0.05) (Fig. 1a).

**Fig. 1.**
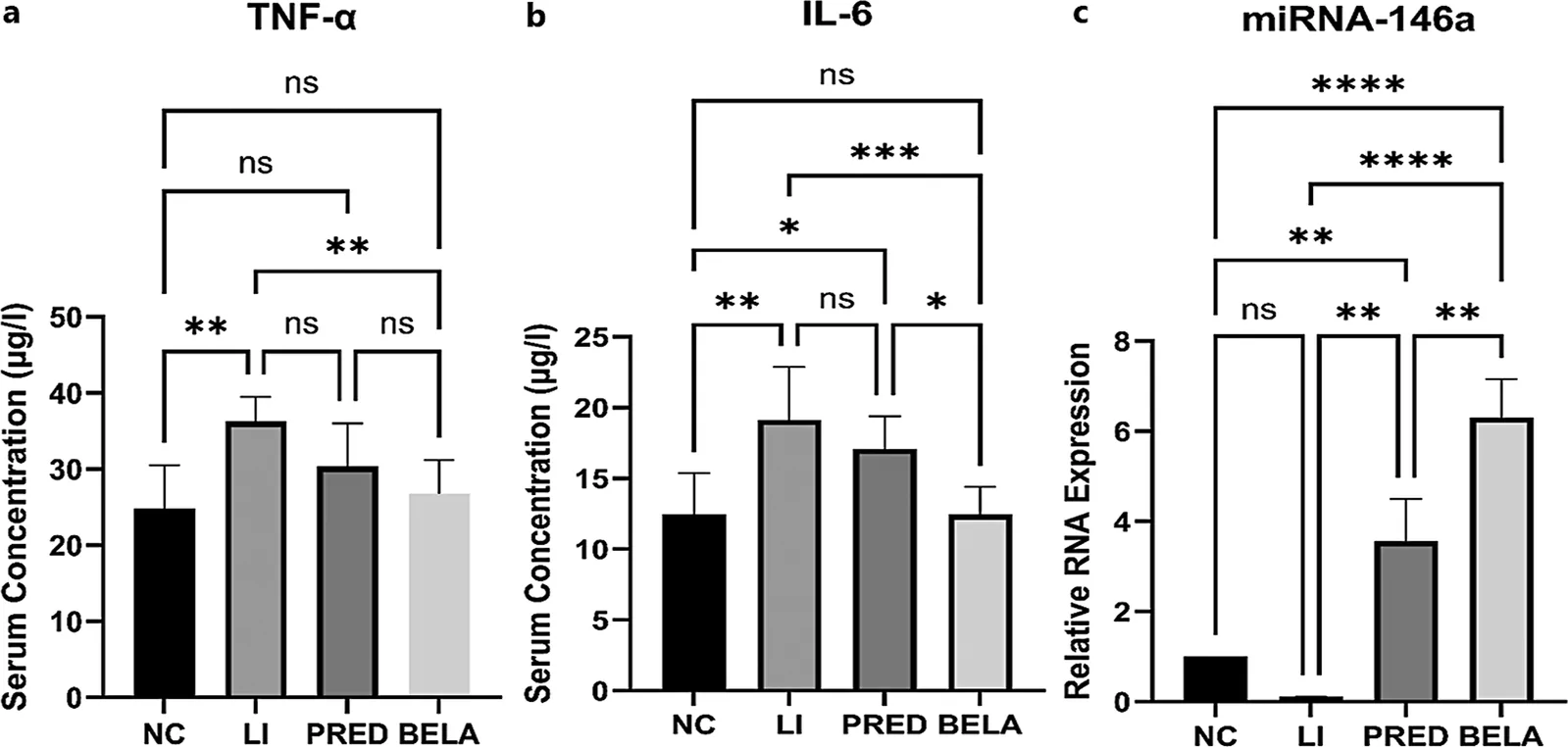
Immunomodulatory effects of Belatacept versus prednisolone on pro-inflammatory markers and microRNA expression. **a** Hepatic TNF-α levels across the experimental groups. **b** Hepatic IL-6 levels across the experimental groups. **c** Relative fold change of miRNA-146a expression normalized to reference control across the experimental groups. NC, normal control group; LI, liver injury group; PRED, prednisolone-treated group; BELA, Belatacept-treated group. Data are presented as mean ± SD (*n* = 6/group), ns: *p* > 0.05; *p < 0.05; **p < 0.01; ***p < 0.001; ****p < 0.0001).

### Belatacept Suppresses IL-6 Production following Hepatic Injury

Serum IL-6 levels peaked significantly on Day 1 post-Con A-induction of hepatic injury, showing a 1.5-fold in the LI group compared to the NC group (*p* < 0.01). Following 14 days of treatment, the improvement in IL-6 levels showed a comparable decline, with significant difference observed between BELA and LI groups (*p* < 0.001). When compared to the LI group, the PRED group showed no statistically significant difference in IL-6 levels. The BELA and PRED groups, however, showed a statistically significant difference (*p* < 0.05). This suggests that belatacept has a stronger systemic immunomodulatory effect than the prednisolone (Fig. 1b).

#### Belatacept Restores miRNA-146a Expression in Hepatic Tissue

To elucidate the molecular mechanism behind this immune suppression, we analyzed the expression of miRNA-146a in hepatic tissue homogenates. The expression of miRNA-146a was downregulated in the LI group. Belatacept treatment effectively upregulate miRNA-146a levels (Fig. 1c), achieving a restoration profile statistically more significant than prednisolone (*p* < 0.01). While bivariate correlation analysis showed a weak inverse relationship between the miRNA-146a expression and the cytokine levels [TNF-α (*r* = −0.212, *p* > 0.05), IL-6 (*r* = −0.394, *p* > 0.05)].

## Discussion

### Main Findings and the “Parallel Recovery Model”

The primary finding of this study is that belatacept significantly attenuates Con A-induced acute liver injury, exhibiting potent immunomodulatory efficacy comparable to prednisolone, the established clinical standard. Specifically, co-stimulatory blockade with belatacept effectively suppressed the systemic cytokine storm (TNF-α and IL-6) while driving a robust restoration of hepatic miRNA-146a expression. To explain these findings, we propose a “Parallel Recovery Model.” In this model, belatacept acts through a dual-action mechanism: first, as a direct signaling inhibitor that rapidly halts TNF-α and IL-6 over secretion; and second, as an epigenetic reprogramming agent that upregulates miRNA-146a expression to serve as a long-term post-transcriptional controller of inflammation.

Mechanistically, Con A-induced hepatic injury has been shown to be driven by the activation of CD4+ T cells, which engage resident hepatic macrophages (Kupffer cells) to release massive amounts of TNF-α and IL-6 [2]. Full activation of these intrahepatic T cells requires the secondary co-stimulatory signal provided by the interaction between CD28 on T cells and CD80/CD86 on antigen-presenting cells. By selectively binding CD80/CD86, belatacept deprives T cells of this critical second signal. This blockade has been shown to affect the hepatic T-cell populations, halting secondary Kupffer cell activation and thus terminate the downstream NF-κB-mediated inflammatory cascade [16, 17].

### Cytokine Suppression, Cellular Targets, and Clinical Perspective

The TNF-α/IL-6 cytokine axis serves as the primary driver of acute hepatic inflammation [18]. Despite the fact that the observed increases in serum TNF-α and IL-6 were modest (less than two times), they were highly statistically significant, indicating a precise and steady inflammatory activation. Since TNF-α is a powerful signal for Kupffer cell activation in the liver, even a small systemic change in it can set off a massive downstream cascade of leukocyte recruitment and inflammation [19].

The potent cytokine suppression by belatacept aligns with previous literature on CTLA4-Ig fusion proteins, which demonstrated marked efficacy in inhibiting cytokines secretion across various systemic inflammatory and transplant rejection models [20, 21]. Our findings demonstrate the possibility of extending belatacept beyond its conventional use in renal transplantation when viewed from a wider therapeutic perspective. Extending CTLA4-Ig treatment to liver injury showed a comparable ability to halt T-cell-mediated tissue destruction [22]. Here, belatacept achieved therapeutic efficacy equivalent to prednisolone, the standard clinical regimen, underscoring its promise as a steroid-sparing alternative for acute immune-mediated liver pathology.

### Epigenetic Reprogramming and Supra-Physiologic Upregulation of miRNA-146a

MiRNAs have been shown to act as “rheostats” to regulate the strength of the innate immune response in the setting of acute hepatic damage [23]. Tumor-necrosis factor receptor-associated factor 6 (TRAF6) is the target of miRNA-146a, which would limit the NF-κB signaling pathway [24, 25]. In the present study, Con A induction caused downregulation of hepatic miRNA-146a. Interestingly, treatment with both prednisolone and belatacept not only restored but markedly induced a supra-physiologic expression of miRNA-146a beyond baseline control levels. Similar supra-physiologic elevations of miRNA-146a have been reported in prior pharmacotherapeutic models, where over-expression functions as an amplified compensatory mechanism to forcefully suppress hyper-inflammatory cascades and accelerate tissue repair [26, 27]. Our Parallel Recovery Model of concurrent cellular and epigenetic regulation is supported by the absence of a simple linear correlation between miRNA-146a expression and cytokine levels, which further demonstrates that belatacept does not depend on a single linear mechanism.

A limitation of the current study is the lack of direct immunophenotyping of liver-infiltrating immune cell populations. Although our findings highlight the inhibition of T-cell-mediated pathways, further research utilizing flow cytometric analysis will be essential to precisely identify which specific intrahepatic leukocyte subsets (e.g., CD4+ T cells, NKT cells, or Kupffer cell populations) are predominantly blocked by belatacept during Con A-induced acute liver injury.

## Conclusion

In hepatic injury, Belatacept can suppress the TNF-α/IL-6 axis. An epigenetic mechanism for its immune-modulation is provided by the restoration of miRNA-146a expression, which at least partially mediates its effectiveness. These findings validate the proposed “Parallel Recovery Model” back up the development of Belatacept as a promising steroid-sparing therapeutic strategy in immune-mediated liver damage. Although these preclinical results are highly promising, current data remain insufficient to support immediate routine clinical application. To confirm belatacept safety, ideal dosage, and therapeutic efficacy in treating acute immune-mediated liver injury in human, further evaluation in Phase I and Phase II clinical trials is necessary.

## Statement of Ethics

All experimental procedures were approved by the Institutional Animal Ethics Committee (College of Pharmacy/Mustansiriyah University, Approval No (62) and performed in compliance with the rules for the handling of experimental animals.

## Conflict of Interest Statement

The authors report no conflicts of interest. The authors alone are responsible for the content and writing of this article.

## Funding Sources

This study was not supported by any sponsor or funder.

## Author Contributions

Doaa Khalid Ibrahim: methodology and writing – original draft; Yassir Mustafa Kamal Al-Mulla Hummadi: supervision; Huda Jaber Waheed: writing – review and editing.

## Data Availability

The data support the findings of this study are available from the corresponding author (D.I.) upon reasonable request.
